# UBE2C triggers HIF‐1α‐glycolytic flux in head and neck squamous cell carcinoma

**DOI:** 10.1111/jcmm.17400

**Published:** 2022-05-26

**Authors:** Yi‐Fang Yang, Yu‐Chan Chang, Kuo‐Wang Tsai, Ming‐Hsin Hung, Bor‐Hwang Kang

**Affiliations:** ^1^ Department of Medical Education and Research Kaohsiung Veterans General Hospital Kaohsiung Taiwan; ^2^ Department of Biomedical Imaging and Radiological Sciences National Yang Ming Chiao Tung University Taipei Taiwan; ^3^ Department of Research, Taipei Tzu Chi Hospital Buddhist Tzu Chi Medical Foundation New Taipei City Taiwan; ^4^ Department of Otorhinolaryngology‐Head and Neck Surgery Kaohsiung Veterans General Hospital Kaohsiung Taiwan; ^5^ Graduate Institute of Aerospace and Undersea Medicine National Defense Medical Center Taipei Taiwan; ^6^ Department of Pharmacy Tajen University Pingtung Taiwan

**Keywords:** glycolysis, HIF‐1α, HNSCCUBE2C

## Abstract

Head and neck squamous cell carcinoma (HNSCC) is the most common malignancy in Taiwan. Therefore, refining the diagnostic sensitivity of biomarkers for early‐stage tumours and identifying therapeutic targets are critical for improving the survival rate of HNSCC patients. Metabolic reprogramming contributes to cancer development and progression. Metabolic pathways, specifically, play a crucial role in these diverse biological and pathological processes, which include cell proliferation, differentiation, apoptosis and carcinogenesis. Here, we investigated the role and potential prognostic value of the ubiquitin‐conjugating enzyme E2 (UBE2) family in HNSCC. Gene expression database analysis followed by tumour comparison with non‐tumour tissue showed that *UBE2C* was upregulated in tumours and was associated with lymph node metastasis in HNSCC patients. Knockdown of UBE2C significantly reduced the invasion/migration abilities of SAS and CAL27 cells. UBE2C modulates glycolysis pathway activation and HIF‐1α expression in SAS and CAL27 cells. CoCl_2_ (HIF‐1α inducer) treatment restored the expression of glycolytic enzymes and the migration/invasion abilities of UBE2C knockdown cells. Based on our findings, UBE2C expression mediates HIF‐1α activation, increasing glycolysis pathway activation and the invasion/migration abilities of cancer cells. UBE2C may be an independent prognostic factor and a therapeutic target in HNSCC.

## 1 INTRODUCTION

Head and neck cancers include cancers of the oral cavity, oropharynx, hypopharynx and larynx. Among the annual deaths in Taiwan in 2019, 50,232 deaths were due to cancers, of which 3425 deaths (14.5%) occurred due to oral cancer (from the Ministry of Health and Welfare of Taiwan). Oral cancer refers to malignancies arising in the intra‐oral sites including the lips, tongue, salivary gland, gingiva, the floor of the mouth, the buccal mucosal surface and others.^1^ Oral squamous cell carcinoma (OSCC) is the most prevalent malignant neoplasm in oral cancer, and its mortality has increased over the past decade.^2^ Approximately 50% of patients with oral cancer have cervical lymph node metastasis at the time of diagnosis and experience poor outcomes.^3^, ^4^, ^5^ Unfortunately, the 5‐year survival rate of patients with oral cancer has not significantly improved despite the availability of multiple treatment modalities, including surgery, radiotherapy, chemotherapy and target therapy.^2^, ^6^, ^7^. Metastasis development in cancer involves multiple steps, in which malignant cells spread from the origin tumour to colonize distant organs.^8^ These basic steps are dependent on the type of cancer. Different cancers have different process steps, signalling pathways and development rates as they colonize distant organs.

Metabolic reprogramming is a major hallmark of cancer and plays an important role in the malignant properties of cancer cells.^9^, ^10^ Moreover, metabolism contributes to the malignant phenotype, signal transduction, tumorigenesis and metastasis of cancer cells.^11^, ^12^, ^13^, ^14^ However, the mechanism by which cancer cells take advantage of bioenergetics to modulate the malignant phenotype has not yet been elucidated.

Ubiquitination is an important cellular mechanism for the degradation of abnormal or short‐lived proteins. The ubiquitin‐conjugating enzyme E2 (UBE2) family is involved in ubiquitination.^15^ In our study, we analysed the members of the UBE2 family that were differentially expressed in patients with head and neck squamous cell carcinoma (HNSCC). We found that *UBE2C* was upregulated in tumour tissues and was associated with poor overall survival in HNSCC. Meanwhile, knockdown of UBE2C inhibited the glycolysis pathway in HNSCC cells, illustrating that UBE2C levels are correlated with the expression of glycolysis enzymes in patients with HNSCC. However, the interplay between UBE2C and the glycolysis pathway in HNSCC development and progression remains elusive.

## 2 MATERIALS AND METHODS

### 2.1 In silico mRNA profiles and Kaplan–Meier analysis of the UBE2 family

The mRNA expression of the UBE2 family was determined using TCGA dataset. Kaplan–Meier analysis (overall survival) was performed using the TCGA dataset (ENCORI, https://starbase.sysu.edu.cn/index.php).

### 2.2 Cell lines and culture conditions

SAS and CAL27 cell lines from human tongue squamous cell carcinoma. CAL27 was purchased from ATCC (#CRL‐2095). SAS was provided from Dr. Michael Hsiao at Academia Sinica in Taiwan. HNSCC cell lines CAL27 and SAS were cultured in DMEM with 10% FBS and 1% PSG.

### 2.3 Lentivirus infection

Lentivirus vector control (pLKO‐1‐shLuc967) and shUBE2C shRNA viral supernatant (TRCN0000004241, TRCN0000368994) were purchased from the National RNAi Core Facility (Taipei, Taiwan; target sequences are provided in Table S3). Viral supernatants used to infect SAS or CAL27 with 8 μg/ml polybrene. After 72 h, cells were selected using 2 μg/ml puromycin.

### 2.4 Growth curve assay

Cells (2000 cells/well for SAS/shluc, SAS/shUBE2C‐1, SAS/shUBE2C‐2, CAL27/shluc, CAL27/shUBE2C‐1 and CAL27/shUBE2C‐1) were seeded in a 96‐well plate for 24–72 h (incubated at 37°C with 5% CO_2_). The cell growth curve was determined using a 3‐(4,5‐dimethylthiazol‐2‐yl)‐2,5‐diphenyltetrazolium bromide (MTT) assay.

### 2.5 Pathway and upstream regulator analysis

We downloaded GSE32873 (as an mRNA microarray of PC3/knockdown UBE2C compared with PC3/knockdown control) [18] and fold change analysis was performed using GEO2R from Gene Expression Omnibus (GEO) website. The UBE2C interaction network pathway and upstream regulator were generated using Ingenuity Pathway Analysis (IPA). To identify upstream regulators related with UBE2C regulation, we analysed differentially expression gene (793 genes from GEO2R) in PC3/knockdown UBE2C compared with PC3/knockdown control and filtered upstream regulator according to z‐score (inhibition, <−2.0). Based on IPA, we identified HIF1A that putatively associated with UBE2C‐knockdown (Table S2).

### 2.6 Cell invasion and migration assay

HNSCC cell migration and invasion capabilities were determined using transwell chambers, as previously described [29]. First, the cells were resuspended in serum‐free medium (2 × 10^5^ cells/ml) and loaded into transwell chambers (upper chamber) in 100 μl. After 48 h, the cells were stained with crystal violet and measured (bottom chamber) under a light microscope.

### 2.7 Colony formation assay

The stable lines were plated in a six‐well plate at a density of 4000 cells/well. After 7 days, the cells were fixed and stained using crystal violet. The number of colonies was counted using the NIH Image J software.

### 2.8 Western blot analyses

A total of 20 μg protein was extracted from SAS (SAS/shLUC, SAS/shUBE2C‐1 and SAS/shUBE2C‐2) and CAL27 (CAL27/shLUC and CAL27/shUBE2C‐1 and CAL27/shUBE2C‐2) cells and loaded onto 15% SDS‐PAGE gel for electrophoresis, and then transferred to PVDF. The antibodies were listed in Table S3.

### 2.9 Quantitative reverse transcription PCR

The stable cell lines SAS/shLUC, SAS/shUBE2C‐1, CAL27/shLUC and CAL27/shUBE2C‐1 (3 × 105 cells/dish) were separately plated in 6‐cm culture plates and incubated overnight at 37°C with 5% CO_2_. The SAS/shUBE2C‐1 and CAL27/shUBE2C‐1 cells were then treated with a CoCl_2_ (HIF‐α inducer) for 48 h, followed by total RNA extraction for quantitative reverse transcription PCR (RT‐qPCR analysis (SYBR system). RT‐qPCR was performed using the appropriate primers (Table S4).

### 2.10 Statistical analysis

A chi‐square test was performed in order to identify the association of UBE2C with sex, tumour stage, tumour size and lymph node status. To identify significant differences between the treatment groups, a two‐tailed Student's *t*‐test was performed. Spearman's correlation was used to determine the correlations between parameters. Statistical significance was set at *p* < 0.05.

## 3 RESULTS

### 3.1 In silico profiles of the UBE2 family members in HNSCC

**FIGURE 1:**
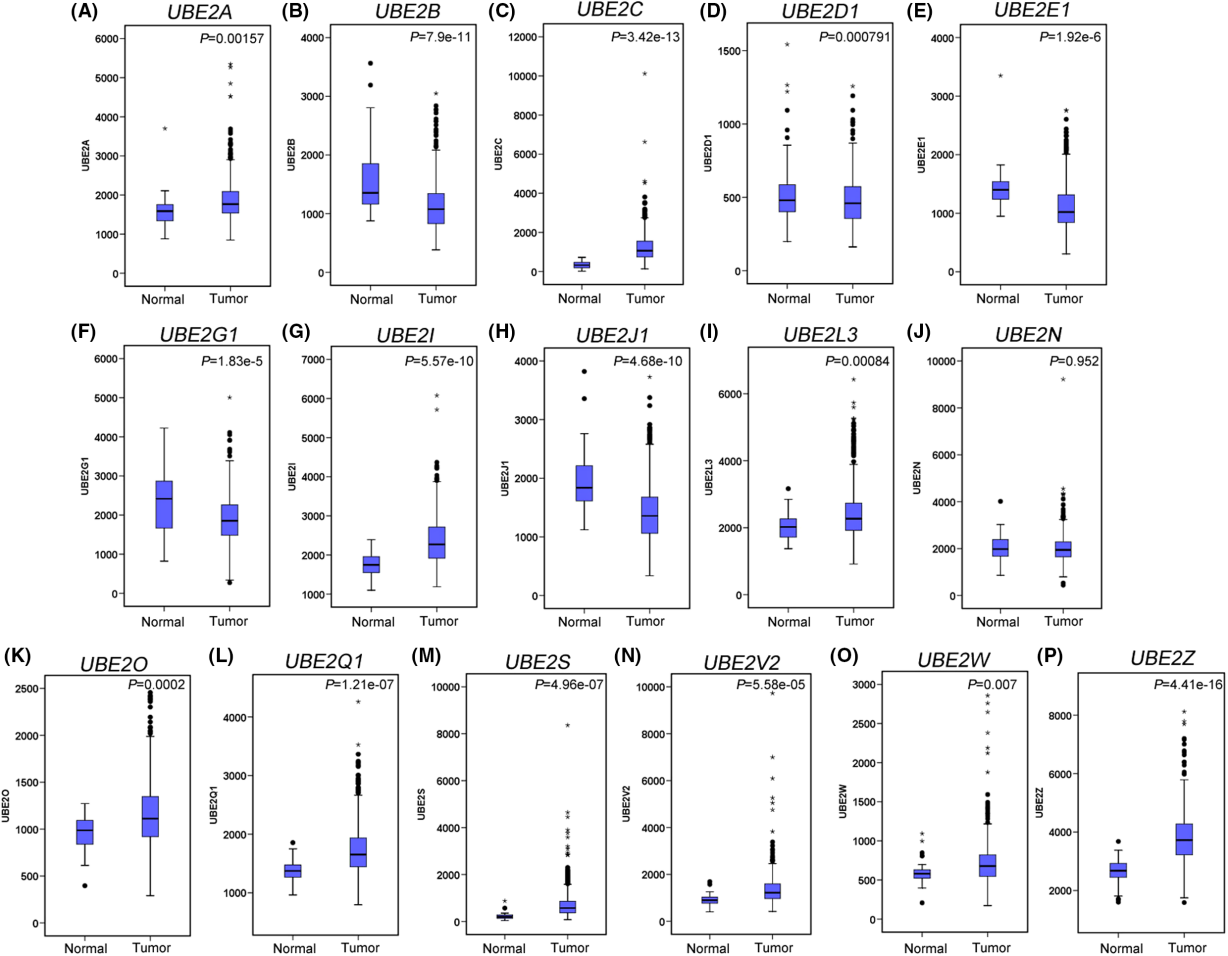
Relative UBE2 family members' mRNA levels in patients with head and neck squamous cell carcinoma (HNSCC), using The Cancer Genome Atlas (TCGA) database. Normal (*n* = 44), Tumour (*n* = 522)

**FIGURE 2:**
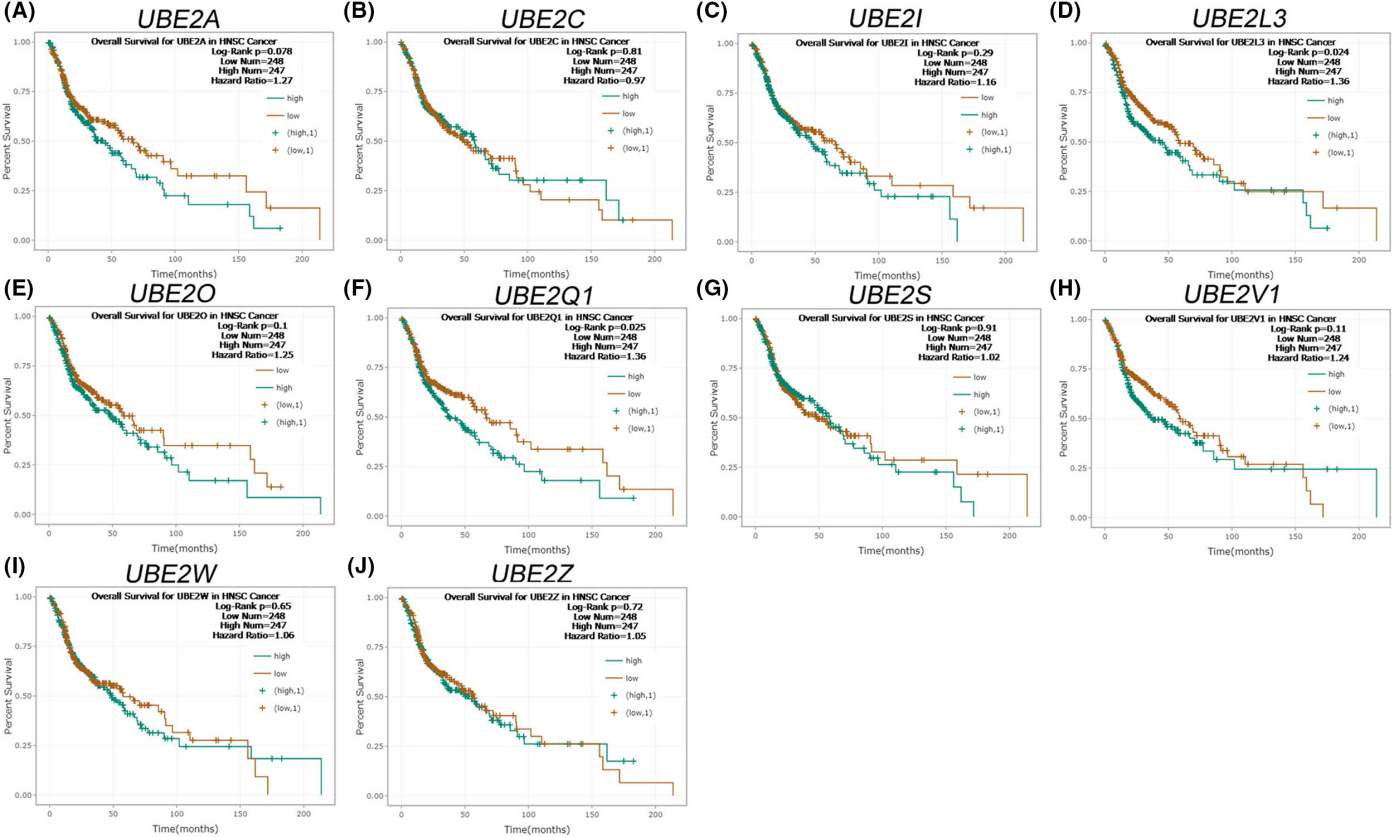
A Kaplan–Meier curve of overall survival, according to the expression of *UBE2A* (A), *UBE2C* (B), *UBE2I* (C), *UBE2L3*(D), *UBE2O* (E), *UBE2Q1* (F), *UBE2S* (G), *UBE2V1* (H), *UBE2W* (I) and *UBE2Z* (J), using an online, publicly available, HNSCC dataset (ENCORI)

We evaluated the expression of the UBE2 family in HNSCC, we used The Cancer Genome Atlas (TCGA) database, including 43 pair and total HNSCC specimens (normal = 44 and tumour = 522). *UBE2A*, *UBE2C*, *UBE2I*, *UBE2L3*, *UBE2O*, *UBE2Q1*, *UBE2S*, *UBE2V2*, *UBE2W* and *UBE2Z* levels were significantly upregulated in patients with HNSCC (Figure 1 and Figure S1). We then examined the overall survival rates of *UBE2A*, *UBE2C*, *UBE2I*, *UBE2L3*, *UBE2O*, *UBE2Q1*, *UBE2S*, *UBE2V2*, *UBE2W* and *UBE2Z* in an online survival analysis. High *UBE2L3* and *UBE2Q1* expression were significantly associated with poor overall survival (Figure 2). Later, we compared *UBE2C*, *UBE2L3* and *UBE2Q1* in transmission organization. *UBE2C* and *UBE2L3* were upregulated in metastatic tissues (Figure S2). In overall survival analysis, UBE2C expression was not significantly associated with overall survival in HNSCC patients. However, previous studies showed that *UBE2C* was upregulated in HNSCC patients and its expression was associated with lymph node metastasis in tongue squamous cell carcinoma patients.^16^, ^17^ Therefore, we focus on UBE2C for further investigation of its molecular mechanism in HNSCC, we used TCGA database. We found that high *UBE2C* levels in patients with HNSCC were significantly associated with sex (*p* = 0.008) and positive lymph node metastasis (*p* = 0.024) (Table 1).

**TABLE 1:** Association of *UBE2C* expression with clinicopathological characteristics in 444 patients with HNSCC

Variables	Item	Patient No.	*UBE2C*		*p* value^*^
			Low	High	
			No. (%)	No. (%)	
		444	118	326	
Sex	Female	122	44	78	0.008
	Male	322	74	248	
Stage	I	27	11	16	0.358
	II	70	19	51	
	III	81	22	59	
	IV	266	66	200	
T status	T1	42	15	27	0.491
	T2	119	35	94	
	T3	98	26	72	
	T4	175	42	133	
Lymph node status	Negative	204	65	139	0.024
	Positive	240	53	187	

^*^ *p* value < 0.05, was considered statistically significant (chi‐square test for categorical variables).

### 3.2 UBE2C knockdown reduced invasion and migration abilities of the HNSCC cells

**FIGURE 3:**
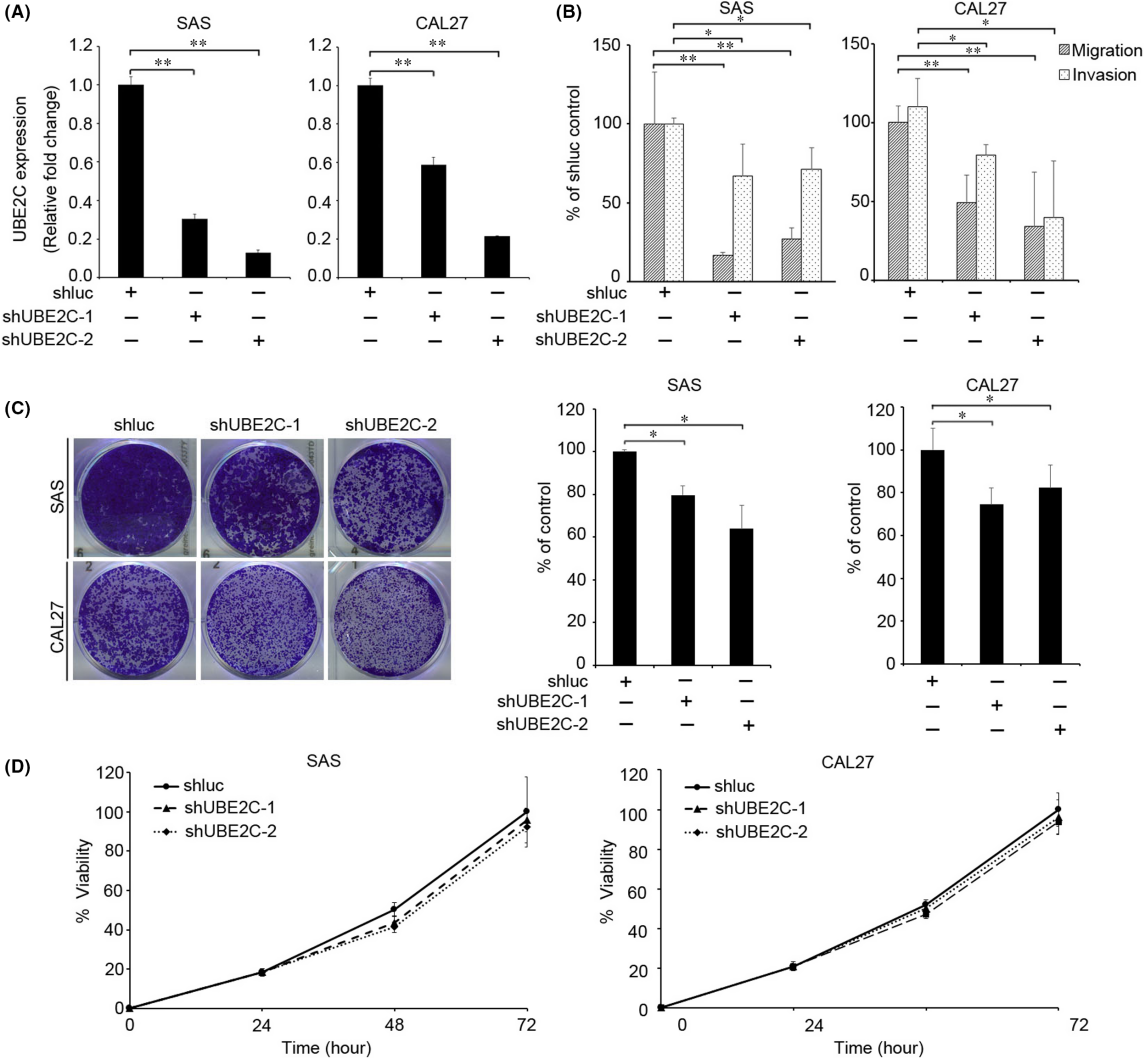
UBE2C downregulation reduced migration, invasion, and colony formation abilities of oral cancer cells. (A) RT‐qPCR analysis of *UBE2C* expression in SAS and CAL27 after virus infection. (B) Effect of UBE2C knockdown on migration and invasion of SAS (left) and CAL27 (right) oral cancer cells. (C) Colony formation assay was performed in SAS (right) and CAL27 cells infected with shluc or shUBE2C. Left: Representative images of the colony formation assays of SAS (Top) and CAL27 (Bottom) cells infected with shluc or two UBE2C shRNAs. Right: Colony formation capabilities of SAS (left) and CAL27 (right) cells following UBE2C knockdown. Data were presented as the mean ± SD; **p < 0.01; *p < 0.05. (D) The proliferation of SAS and CAL27 cells infected with shUBE2C or shluc shRNA were analysed using the MTT assay (*n* = 8)

Clinical findings suggest that UBE2C plays a role in HNSCC progression. We evaluated the effects of UBE2C knockdown on the migration, invasion, colony formation and proliferation abilities of oral cancer cells. As shown in Figure 3A, the expression levels of UBE2C decreased after silencing via lentiviral‐mediated RNAi. Knockdown of UBE2C expression in SAS and CAL27 HNSCC cell lines significantly reduced their migration, invasion and colony formation abilities compared with shluc control cells (Figure 3B,C and Figure S3). Moreover, knockdown of *UBE2C* did not affect the viability of SAS and CAL27 cells (Figure 3D).

### 3.3 UBE2C downregulated the inhibitory glycolysis pathway in the HNSCC cells

**FIGURE 4:**
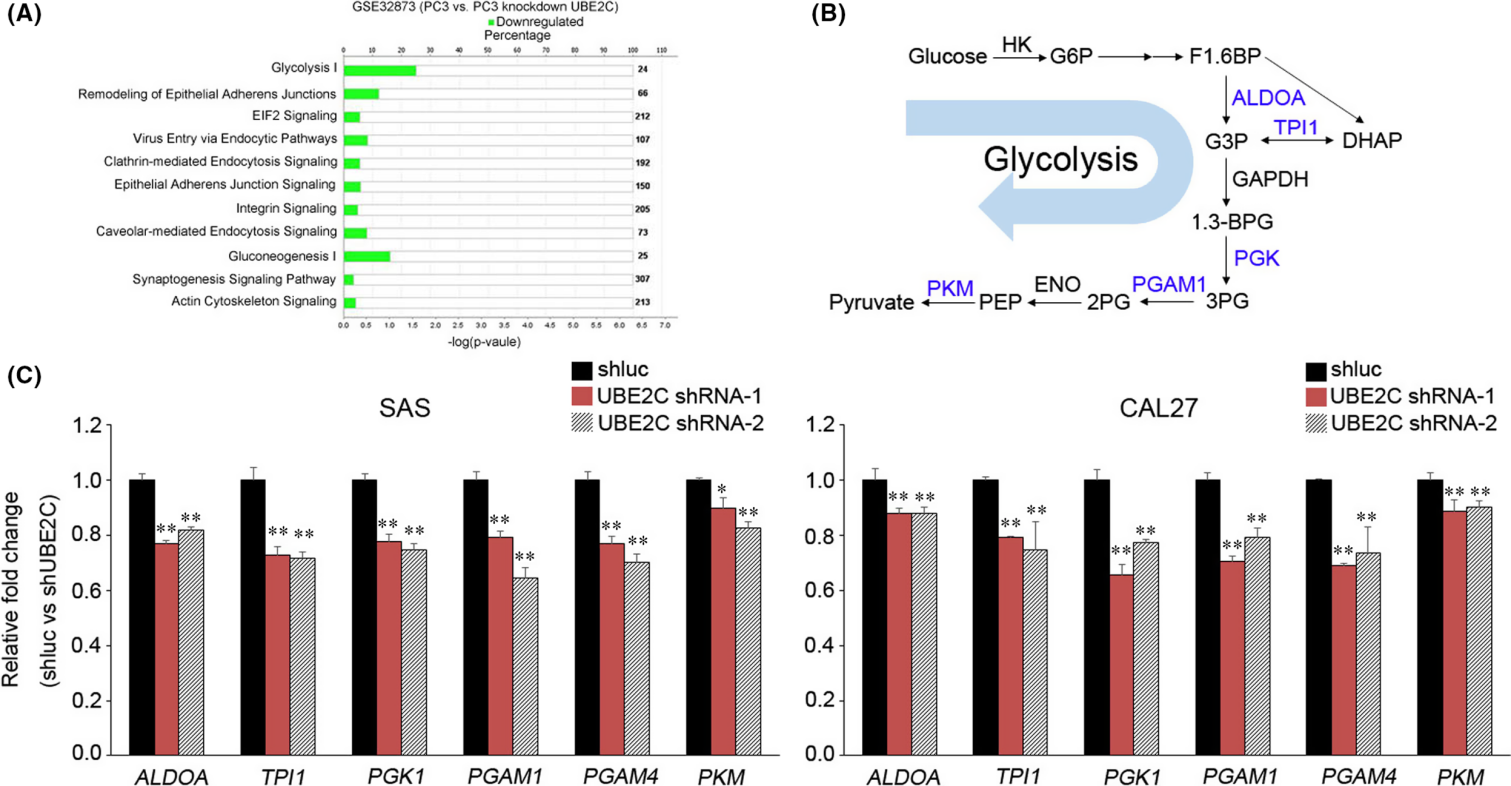
Knockdown of UBE2C resulted in the downregulation of the glycolysis pathway. (A) Pathway analysis of genes differentially expression between control and knockdown UBE2C PC3 cells (GSE32873). (B) Schematic diagram of the inhibited (blue) glycolysis pathway in a UBE2C knockdown cell. (C) RT‐qPCR analysis of *ALDOA*, *TPI1*, *PGK1*, *PGAM1*, *PGAM4* and *PKM* expression in UBE2C knockdown cells and shluc control cells. Data were presented as the mean ± SD; **p < 0.01; *p < 0.05. ns, not significant

To determine the molecular mechanism of the metabolic pathway by which UBE2C regulates cancer cell progression, we downloaded GSE32873 and analysed differential gene expression between control PC3 cells and UBE2C‐knockdown PC3 cells.^18^ By filtering the gene signature enzyme annotation in addition to ingenuity pathway analysis (IPA), the glycolysis I pathway was significantly downregulated in UBE2C‐knockdown cancer cells (Figure 4A and Table S1). Six gene‐encoding enzymes involved in the glycolysis biosynthetic pathway were downregulated in UBE2C knockdown cells: (1) aldolase, fructose‐bisphosphate A (*ALDOA*), (2) triosephosphate isomerase 1 (*TPI1*), (3) phosphoglycerate kinase 1 (*PGK1*), (4) phosphoglycerate mutase 1 (*PGAM1*), (5) phosphoglycerate mutase family member 4 (*PGAM4*) and (6) pyruvate kinase M1/2 (*PKM*) (Figure 4B). We next examined whether UBE2C modulated the glycolysis pathway in SAS and CAL27 cells. We confirmed these results using quantitative reverse transcription polymerase chain reaction (RT‐qPCR), which showed that *ALDOA*, *TPI1*, *PGK1*, *PGAM1*, *PGAM4* and *PKM* were significantly downregulated in UBE2C‐knockdown SAS and CAL27 cells compared with the control cells (Figure 4C). Moreover, UBE2C knockdown significantly reduced lactate levels in HNSCC cells (Figure S4).

### 3.4 *UBE2C* expression correlated with the enzymes of a glycolysis pathway in patients with HNSCC

**FIGURE 5:**
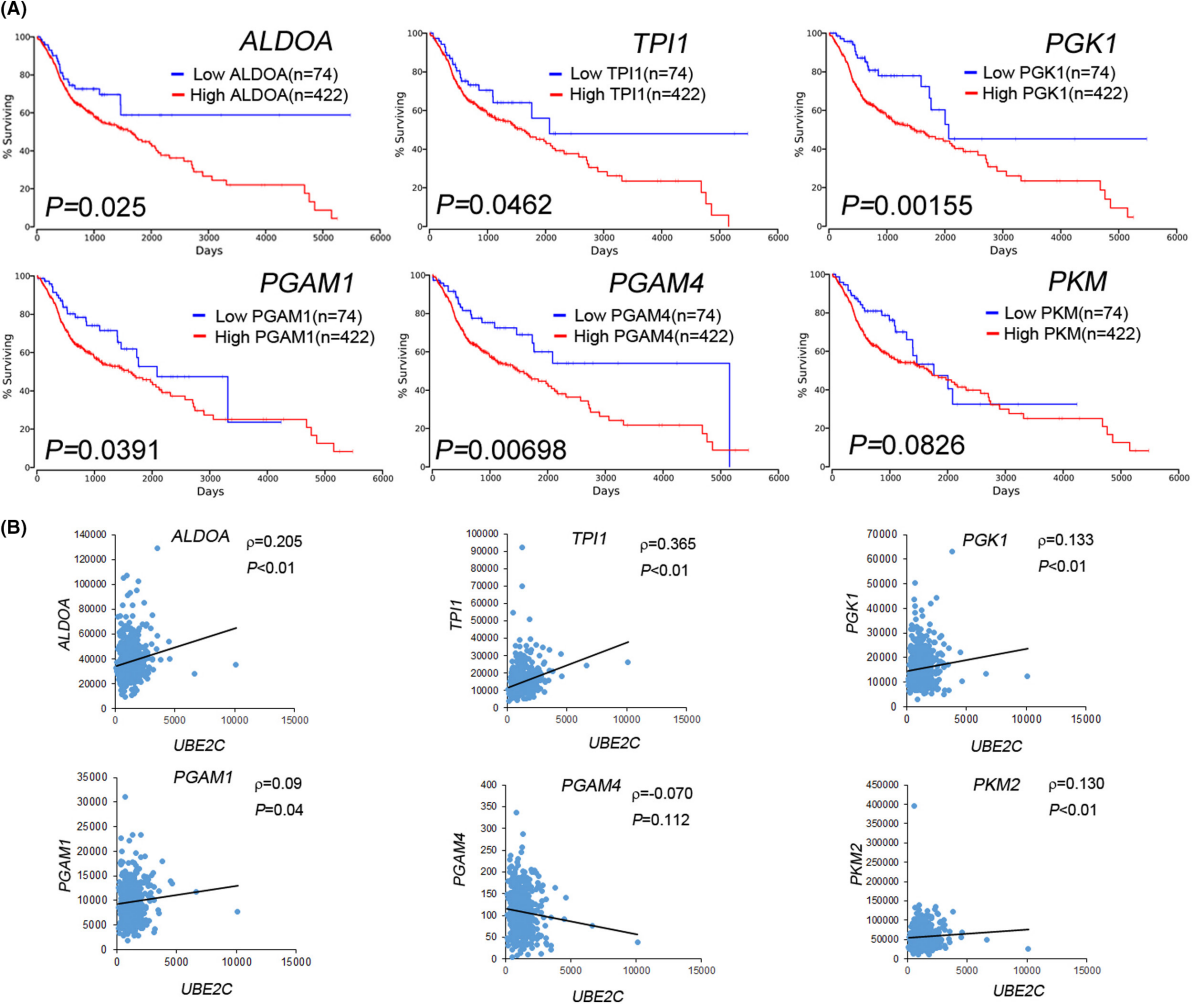
*UBE2C* expression is correlated with the enzymes of the glycolysis pathway in patients with HNSCC. (A) Kaplan–Meier analysis of overall survival, according to the expression of *ALDOA*, *TPI1*, *PGK1*, *PGAM1*, *PGAM4* and *PKM* using an online HNSCC dataset (OncoLnc). (B) Representation of *UBE2C* correlation with *ALDOA*, *TPI1*, *PGK1*, *PGAM1*, *PGAM4* and *PKM* in patients with HNSCC

We next examined the overall survival rates of *ALDOA*, *TPI1*, *PGK1*, *PGAM1*, *PGAM4* and *PKM* via an online survival analysis of patients with HNSCC (http://www.oncolnc.org/). High levels of *ALDOA* (*p* = 0.025), *TPI1* (*p* = 0.0462), *PGK1* (*p* = 0.00155), *PGAM1* (*p* = 0.0391) and *PGAM4* (*p* = 0.00698) were associated with poor overall survival (Figure 5A). We further examined the correlation between *UBE2C* and genes of the glycolysis I pathway in patients with HNSCC using the TCGA dataset. We found *that UBE2C* levels were significantly positively correlated with *ALDOA*, *TPI1*, *PGK1*, *PGAM1* and *PKM2* levels in patients with HNSCC (Figure 5B).

### 3.5 UBE2C promotes cell migration/invasion through the hypoxia inducible factor 1 subunit alpha (HIF‐1α) signalling pathway

**FIGURE 6:**
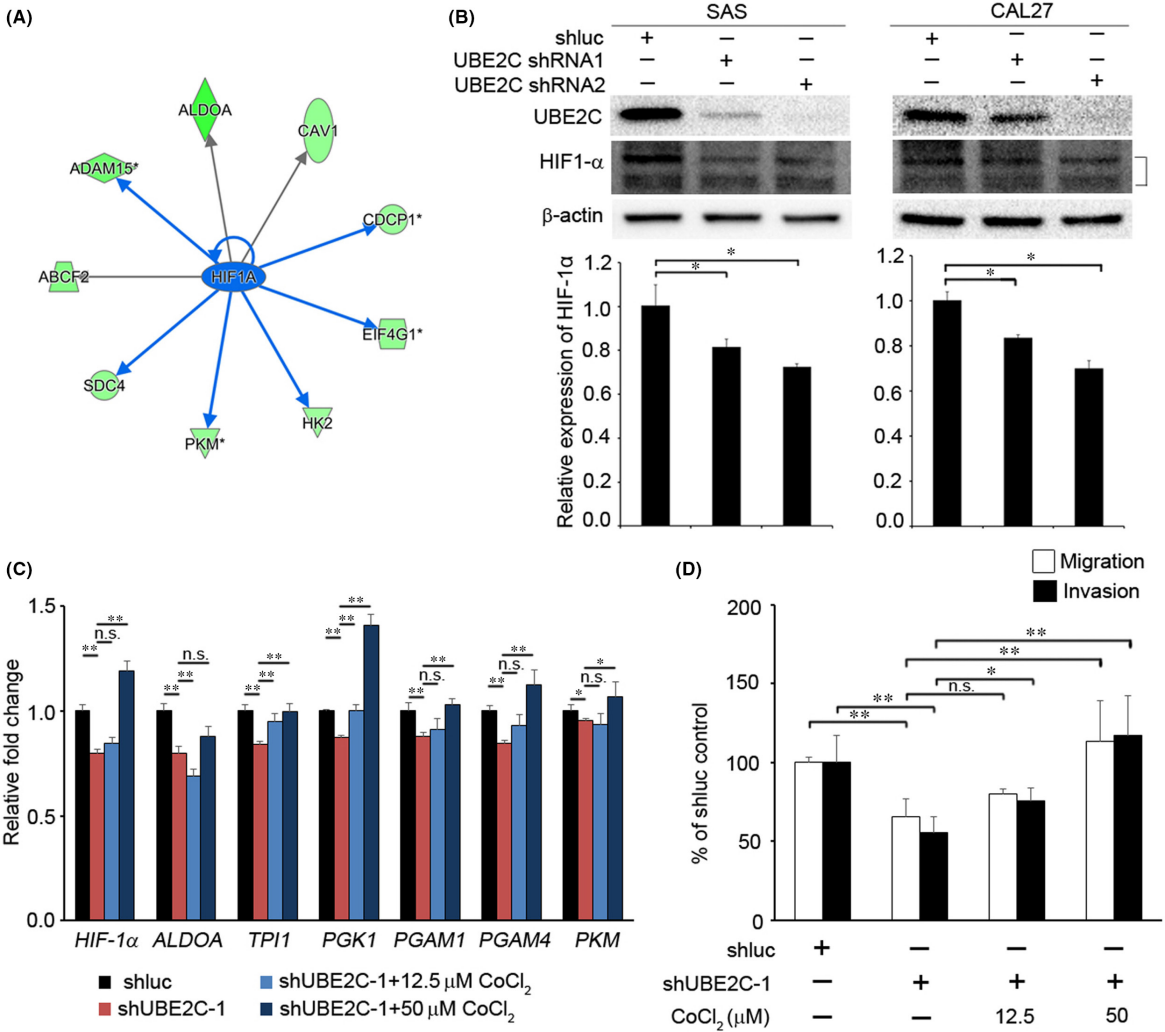
UBE2C modulates invasion/migration through HIF‐1α signalling. (A) Differentially regulated HIF‐1α targets genes following UBE2C knockdown in PC3 cells. The colour (green) indicates the degree of downregulation following UBE2C knockdown. (B) Relative expression of *HIF‐1α* in SAS and CAL27 cells after UBE2C knockdown. *Top*, western blot analysis; *Bottom*, RT‐qPCR. (C) CAL27/shUBE2C cells were treated with CoCl_2_ (as HIF‐1α inducer) for 48 h and analysed by RT‐qPCR. (D) Treatment with or without CoCl_2_ and migration/invasion potential of CAL27/shUBE2C cells. Data are presented as the mean ± SD. *p < 0.05; **p < 0.01

Based on GSE32873 (Figure 4A), a gene annotation enrichment analysis was performed to identify transcription factors which showed that HIF‐1α signalling was inhibited upon UBE2C‐knockdown (Figure 6A and Table S2). It has been demonstrated that HIF‐1α regulation glycolysis occurs in cancer cells.^19^ We confirmed whether UBE2C modulated the expression of HIF‐1α in HNSCC cells. Knockdown of UBE2C in CAL27 and SAS significantly reduced HIF‐1α expression (Figure 6B). Next, we evaluated whether UBE2C mediated migration/invasion and colony formation capabilities in UBE2C knockdown cells. Treatment with CoCl_2_ (a HIF‐1α inducer) restored the expression of *HIF‐1α*, *TPI1*, *PGK1*, *PGAM1*, *PGAM4* and *PKM*, as well as the migration/invasion capabilities in the UBE2C knockdown cells (Figure 6C,D and Figure S5). These data suggest that UBE2C enhances cell migration and invasion abilities through HIF‐1α signalling.

## 4 DISCUSSION

In this study, the UBE2 family was assessed in patients with HNSCC, and high levels of *UBE2C* were identified as a signifier for poor prognosis in patients. High levels of *UBE2C* expression were also correlated with lymph node metastasis, indicating that the enzyme plays an important role in HNSCC metastasis. Although other members of the UBE2 family have been implicated for their role in cancer metastasis, the UBE2O‐AMPKα2 axis, for example, was found to promote breast cancer metastasis.^20^ We have shown that these members are unrelated to the poor survival of patients with HNSCC.

An earlier study reported that the von Hippel–Lindau protein (pVHL) is responsible for modulating HIF‐1α expression ^21^, ^22^, ^23^, ^24^ A separate study described how UBE2C regulates HIF‐1α expression by ubiquitinating and degrading pVHL (an upstream regulator of HIF‐1α) in human aortic valve endothelial cells.^25^ In the present study, knockdown of UBE2C significantly reduced the invasion/migration and colony formation abilities of HNSCC cells (Figure 3B,C). Our data illustrated that UBE2C mediates the invasion and migration capabilities through the HIF‐1α pathway in HNSCC cells. In agreement with our results, Nora et al. showed that CoCl_2_ treatment significantly increased cell motility in YS1.2 and pII cells.^26^ Meanwhile, our data showed that treatment with CoCl_2_ restored the invasion/migration abilities in UBE2C knockdown cells (Figure 6D). These findings suggest that UBE2C‐HIF‐1α signalling promotes HNSCC cell invasion.

Furthermore, we showed that UBE2C mediated both the glycolysis pathway and the expression of HIF‐1α in HNSCC cells (Figures 4C and 6B). Previous studies have shown that HIF‐1α is involved in regulating the glycolysis pathway. In hypoxic stimuli, HIF‐1α‐induced glucose transporter 1 (GLUT1) expression increases glucose uptake and supports glycolysis in cancer cells.^27^ HIF‐1α was also found to both modulate ALDOA expression and increase lactate levels, leading to upregulation of MMP9, thereby promoting an invasion of lung cancer cells in vitro and in vivo.^28^ Similarly, our data showed that UBE2C regulated the expression of HIF‐1α and glycolysis enzymes in HNSCC cells. Knockdown of UBE2C reduced lactate levels in HNSCC cells. CoCl_2_‐induced HIF‐1α expression restored migration and invasion abilities as well as the glycolysis pathway in HNSCC cells. Based on these findings, we surmised that HIF‐1α is regulated by UBE2C, resulting in both a regulated glycolysis pathway as well as the advancement of the HNSCC cells' invasion ability.

Other mechanistic pathways have been suggested for UBE2C. In a previous study, treatment with CCI779 (mTORC1 complex inhibitor) inhibited UBE2C expression and tumour growth in cervical cancer in vitro and in vivo.^29^ UBE2O, a UBE2 member, targets AMPKα2 for ubiquitination and degradation, leading to an increase in the mTORC1‐HIF‐1α pathway. UBE2O modulates cancer progression through the AMPKα2‐mTORC1‐HIF‐1α axis in cancer cells. Moreover, inhibition of UBE2O reduced glucose consumption and lactate levels in HAP1 cells.^20^ Our data showed that UBE2C regulated both the glycolysis pathway and invasion ability through HIF‐1α signalling in HNSCC cells.

Metabolic reprogramming is a hallmark of cancer and is implicated in cancer progression contributing to signal transduction and metastasis.^9^, ^28^ High glucose levels generate lactate secretion into the extracellular space, which may create a tumour microenvironment favourable for cancer cell migration and angiogenesis.^30^ Moreover, HIF‐1α‐modulated ALDOA upregulated expression in lung cancer cells. Meanwhile, ALDOA overexpression promoted lactate production in order to block prolyl hydroxylase (PHD) activities, leading to HIF‐1α stabilization, thereby promoting metastasis.^28^ Our data showed that knockdown of UBE2C reduced the expression of *ALDOA* in HNSCC cells. *UBE2C*‐upregulated expression correlates with *ALDOA* expression in patients with HNSCC. High ALDOA expression was also associated with poor overall survival. In this study, we demonstrated that UBE2C mediates the glycolysis pathway and invasion ability, thereby providing a therapeutic target for HNSCC metastasis.

## 5 CONCLUSION

After evaluating the expression of the UBE2 family in this study, upregulated *UBE2C* was identified to be associated with lymph node metastasis in patients with HNSCC. Moreover, this study is the first to investigate the mechanism by which UBE2C modulates the migration/invasion abilities and glycolysis pathway of HNSCC cells through HIF1‐α signalling. Here, we found that *UBE2C* expression was correlated with lymph node metastasis in patients with HNSCC, indicating that UBE2C is an independent prognostic factor.

## AUTHOR CONTRIBUTIONS

**Yi‐Fang Yang:** Formal analysis (equal); funding acquisition (equal); writing – original draft (equal). **Yu‐Chan Chang:** Funding acquisition (equal); validation (equal). **Kuo‐Wang Tsai:** Resources (equal). **Ming‐Hsin Hung:** Data curation (equal). **Bor‐Hwang Kang:** Funding acquisition (equal); writing – review and editing (equal).

## CONFLICT OF INTEREST

The authors declare that they have no conflict of interest.

## Supporting information


**Appendix S1** Supporting Information

## Data Availability

The datasets used and/or analysed during the current study are available from the corresponding author on reasonable request.
