# Supplementary material for: UBE2C triggers HIF‐1α‐glycolytic flux in head and neck squamous cell carcinoma
Source: J Cell Mol Med. 2022 May 26;26(13):3716–25. doi: 10.1111/jcmm.17400 (PMC9258705; doi:10.1111/jcmm.17400)
Supplement: Supplementary file 1 — Appendix S1 Supporting Information [file JCMM-26-3716-s001.docx]

**UBE2C triggers HIF-1α-glycolytic flux in head and neck squamous cell carcinoma**

Yi-Fang Yang^1^ **iD**, Yu-Chan Chang^2^ **iD**, Kuo-Wang Tsai ^3^ **iD**, Ming-Hsin Hung^1^, and Bor-Hwang Kang^4,5,6^* **iD**

https://orcid.org/0000-0001-7425-3156 (Yi-Fang Yang)

https://orcid.org/0000-0003-0474-9935 (Yu-Chan Chang)

https://orcid.org/0000-0002-9028-9834 (Kuo-Wang Tsai)

https://orcid.org/0000-0003-3788-448X (Bor-Hwang Kang)

^1^ Department of Medical Education and Research, Kaohsiung Veterans General Hospital, Kaohsiung, Taiwan

^2^ Department of Biomedical Imaging and Radiological Sciences, National Yang Ming Chiao Tung University, Taipei, Taiwan

^3^ Department of Research, Taipei Tzu Chi Hospital, Buddhist Tzu Chi Medical Foundation, New Taipei City, Taiwan

^4^ Department of Otorhinolaryngology-Head and Neck Surgery, Kaohsiung Veterans General Hospital, Kaohsiung, Taiwan

^5^ Graduate Institute of Aerospace and Undersea Medicine, National Defense Medical Center, Taipei, Taiwan

^6^ Department of Pharmacy, Tajen University, Pingtung, Taiwan

Correspondence to: Bor-Hwang Kang, M.D., PhD.

Department of Otorhinolaryngology-Head and Neck Surgery, Kaohsiung Veterans General Hospital, Kaohsiung, Taiwan, No. 386, Dajhong 1st Rd., Zuoying Dist., Kaohsiung City 81362, Taiwan

Phone: 886-7-342-2121 # #70255; Fax: 886-7-342-2288; E-mail: bhkang@vghks.gov.tw

**Conflict of interest:** The authors declare that they have no conflict of interest.

Inventory of all Supplemental Information

Supplemental Data

Figure S1 Related to Figure 1

Figure S2 Related to Figure 2

Figure S3 Related to Figure 3

Figure S4 Related to Figure 4

Figure S5 Related to Figure 6

Supplementary Tables

Supplementary Table 1 Related to Figure 4

Supplementary Table 2 Related to Figure 6

Supplementary Table 3 Key resources table

Supplementary Table 4 Primer sequence

**Supplemental materials and methods**

**Lactate assay**

CAL27/shluc and CAL27/shUBE2C-1 cells were plated at 3 × 10^5^ cells in a 6-well plate for 48 h at 37°C. After incubation, the conditioned medium was collected. Lactate was measured using the Amplite™ Colorimetric L- Lactic acid (L-Lactate) Assay Kit (AAT Bioquest, # 13815).


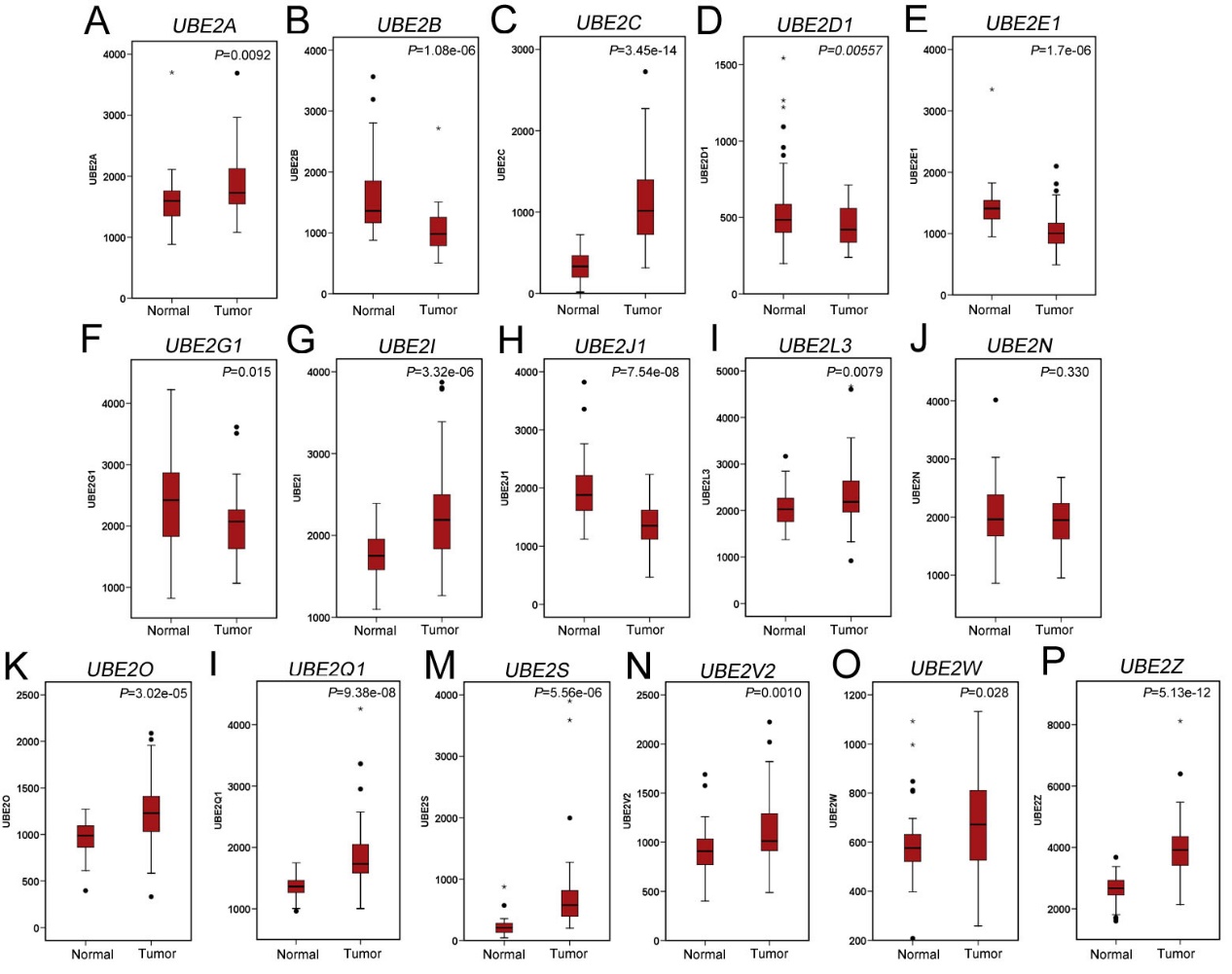


**Supplemental Figure 1 (related to Figure 1).** Quantification of UBE2 family expression in 43 paired HNSCC specimens. *P*-value was determined by Student *t*-test.


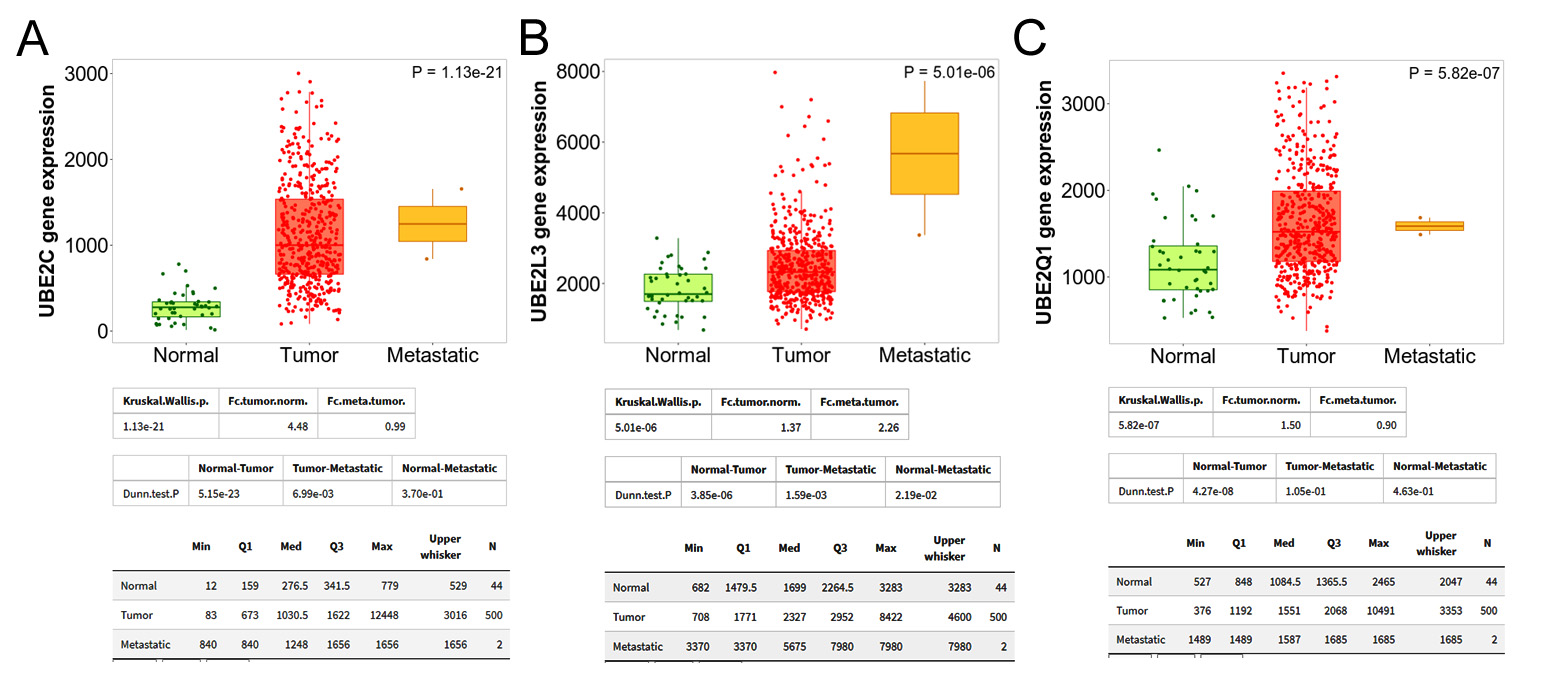


**Supplemental Figure 2 (related to Figure 2).** Quantification of UBE2C, UBE2L3, and UBE2Q1 expression in normal, tumor, and metastatic tissues with HNSCC (TNMplot).


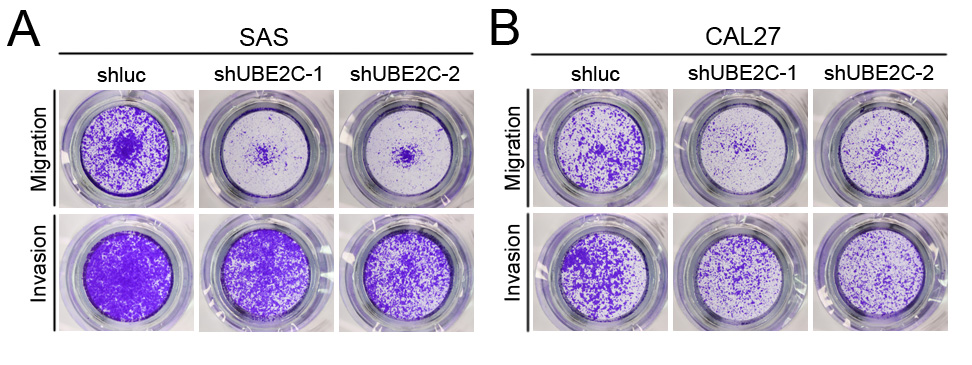


**Supplemental Figure S3 (related to Figure 3). Knockdown of UBE2C inhibits invasion and migration of HNSCC cell lines.**

Representative images of the migration/invasion assays of SAS (A) and CAL27 (B) cells infected with shluc or two UBE2C shRNAs.


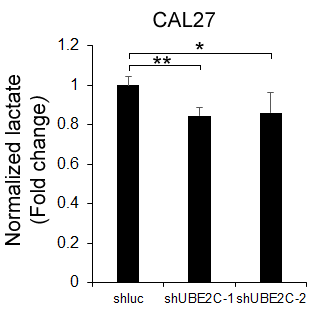


**Supplemental Figure S4 (related to Figure 4). Knockdown of UBE2C reduced lactate production in HNSCC cell lines.**

Changes in lactate levels in the conditioned medium of CAL27 cells infected with shluc, UBE2C shRNA-1, and UBE2C shRNA-2. Data are presented as the mean ± SD. ***P* < 0.01.


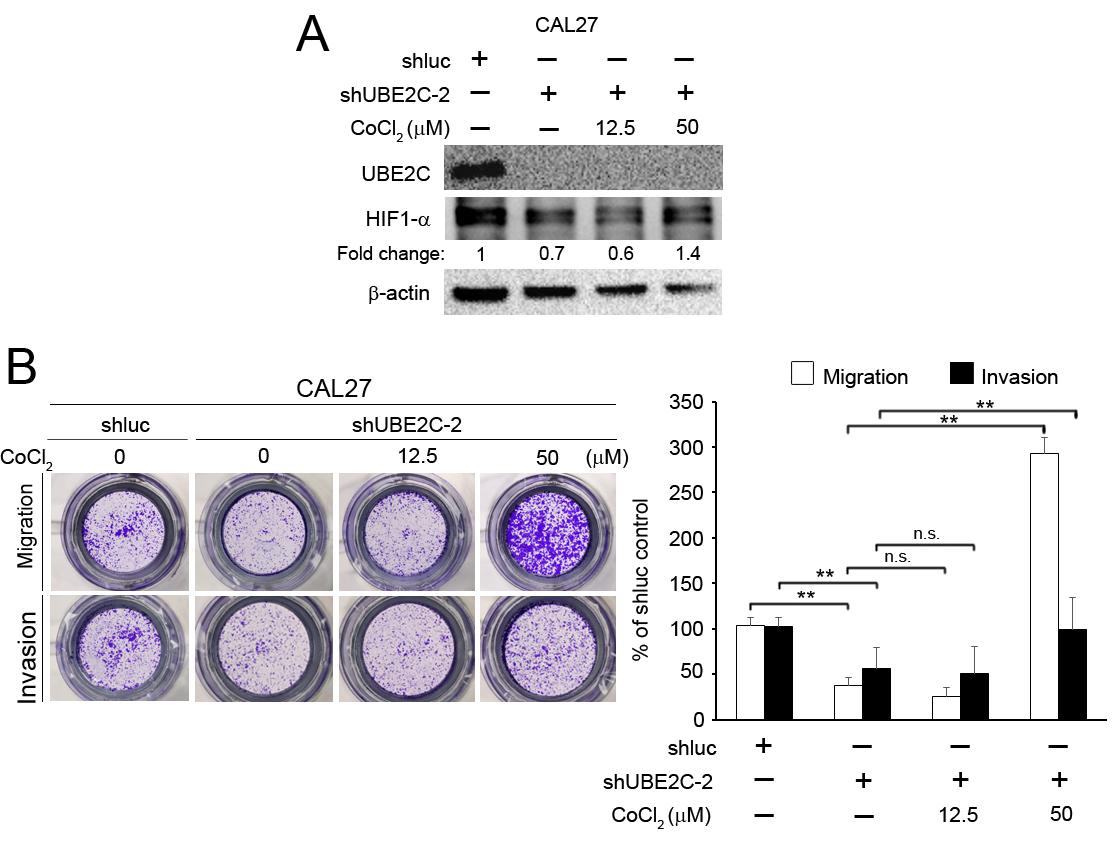


**Supplemental Figure 5 (related to Figure 6). CoCl_2_ restored migration and invasion abilities in CAL27 cells.** (A) CAL27/shUBE2C cells were treated with CoCl_2_ (as HIF-1α inducer) for 48 h and analyzed by western blot. (B) Left, representative images of the migration/invasion assays of CAL27 cells infected with shluc or UBE2C shRNA 2. *P*-value was determined by Student *t*-test.

Supplement Table1. Ingenuity Canonical Pathways

| **Ingenuity Canonical Pathways** | **-log(p-value)** | **z-score** | **Molecules** |
| --- | --- | --- | --- |
| Integrin Signaling | 5.08E+00 | -2.828 | ACTB, ACTG1, ARPC1B, ARPC4, BCAR1, CAPNS1, CAV1, CRK, MPRIP, TSPAN4 |
| Synaptogenesis Signaling Pathway | 4.28E+00 | -2.714 | AP2A1, AP2B1, AP2S1, ARPC1B, ARPC4, BAD, CRK, GPAA1, GRINA,RPS6KB2,STX1A |
| Glycolysis I | 7.45E+00 | -2.449 | ALDOA,PGAM1,PGAM4,PGK1,PKM,TPI1 |
| Fc Receptor-mediated Phagocytosis in Macrophages and Monocytes | 3.91E+00 | -2.449 | ACTB,ACTG1,ARPC1B,ARPC4,CRK,RPS6KB2 |
| EIF2 Signaling | 6.68E+00 | -2.236 | ACTB, EIF3B, EIF4G1, EIF5, PPP1CA, RPL10A, RPL29, RPL8,  RPLP0,RPS2,RPS4X,RPS9 |
| NRF2-mediated Oxidative Stress Response | 3.03E+00 | -2.236 | ACTB,ACTG1,CCT7,CDC34,FTL,STIP1,UBB |
| Sirtuin Signaling Pathway | 2.60E+00 | -2.236 | APP,NR1H2,PGAM1,PGAM4,PGK1,TSPO,TUBA1A,TUBA1C |
| Reelin Signaling in Neurons | 2.37E+00 | -2.236 | APP,ARPC1B,ARPC4,CRK,GRINA |
| Ephrin Receptor Signaling | 1.79E+00 | -2.236 | ARPC1B,ARPC4,BCAR1,CRK,GRINA |
| Signaling by Rho Family GTPases | 1.30E+00 | -2.236 | ACTB,ACTG1,ARPC1B,ARPC4,PKN1 |
| Actin Cytoskeleton Signaling | 4.13E+00 | -2.121 | ACTB,ACTG1,ARPC1B,ARPC4,BCAR1,CRK,FLNA,MPRIP,WASF2 |
| Opioid Signaling Pathway | 3.00E+00 | -2.121 | AP2A1,AP2B1,AP2S1,BAD,GRINA,RPS6KA4,RPS6KB2,SIGMAR1 |
| Gluconeogenesis I | 4.30E+00 | -2 | ALDOA,PGAM1,PGAM4,PGK1 |
| Paxillin Signaling | 1.93E+00 | -2 | ACTB,ACTG1,BCAR1,CRK |
| Leukocyte Extravasation Signaling | 1.11E+00 | -2 | ACTB,ACTG1,BCAR1,CRK |
| Senescence Pathway | 7.24E-01 | -2 | CAPNS1,HIPK2,PPP1CA,RPS6KA4 |
| RhoA Signaling | 3.29E+00 | -1.633 | ACTB,ACTG1,ARPC1B,ARPC4,MPRIP,PKN1 |
| Regulation of Actin-based Motility by Rho | 2.21E+00 | -1 | ACTB,ARPC1B,ARPC4,MPRIP |
| Insulin Receptor Signaling | 1.58E+00 | -1 | BAD,CRK,PPP1CA,RPS6KB2 |
| ILK Signaling | 1.17E+00 | -1 | ACTB,ACTG1,FLNA,RPS6KA4 |
| ERK/MAPK Signaling | 1.66E+00 | -0.447 | BAD,BCAR1,CRK,PPP1CA,RPS6KA4 |
| PTEN Signaling | 2.40E+00 | 1.342 | BAD,BCAR1,CSNK2A1,RPS6KB2,SHARPIN |
| RhoGDI Signaling | 1.79E+00 | 2.236 | ACTB,ACTG1,ARPC1B,ARPC4,WASF2 |

Supplement Table2. Up-stream regulator

| **Upstream Regulator** | **Molecule Type** | **Predicted Activation State** | **Activation z-score** | **p-value of overlap** | **Target Molecules in Dataset** |
| --- | --- | --- | --- | --- | --- |
| ERBB2 | kinase | Inhibited | -2.646 | 1.12E-02 | CDCP1,CHD4,LTBP3,MCM3,PGK1,SMTN,TUBA1A |
| IL15 | cytokine | Inhibited | -2.449 | 3.36E-04 | ALDOA,HK2,PGAM4,PGK1,PKM,TPI1 |
| HIF1A | transcription regulator | Inhibited | -2.418 | 9.15E-05 | ABCF2,ADAM15,ALDOA,CAV1,CDCP1,EIF4G1,HK2,PKM,SDC4 |
| MYC | transcription regulator | Inhibited | -2.407 | 9.97E-04 | CAV1,CD151,CDC34,EIF4G1,HK2,HSPD1,ST3GAL1,TPI1,UBE2C |
| EHMT1 | transcription regulator | Inhibited | -2 | 9.47E-03 | ANPEP,ATP1A1,GRINA,ST3GAL1 |
| SYVN1 | transporter |  | -1.673 | 1.27E-04 | APP,ATP1A1,CD151,CRTAP,CYB561,MYOF,TLE5 |
| PCGEM1 | other |  | -1.446 | 2.50E-05 | ALDOA,CS,HK2,PGK1,PKM |
| ESRRG | ligand-dependent nuclear receptor |  | -1.199 | 1.73E-05 | ACO2,CS,HK2,PKM,TPI1 |
| TCR | complex |  | -1.195 | 4.25E-07 | ALDOA, CS, FLNA, HSPD1, PGAM1, PGK1, RPL10A, RPLP0, RPS2, RPS4X,TNFRSF12A,TPI1 |
| CLDN7 | other |  | 0 | 1.86E-02 | GRINA,H2AC18/H2AC19,PRNP,RPL29 |
| CST5 | other |  | 0.447 | 2.83E-02 | CAV1,DDX21,EEF1D,EEF1G,EMP3,PYGB |
| TP53 | transcription regulator |  | 0.718 | 7.96E-05 | ACTB, ALDH1A3, ARPC1B, CAV1, CSNK1D, CTSD, DNM2, H2AX, HK2, MCM3, MTA1, PPP1CA, PRNP, RALY, TAGLN2, TUBB, UBE2C |
| MRTFA | transcription regulator |  | 1 | 1.78E-03 | ACTB, ARPC1B, ARPC4, CRK |
| HSF1 | transcription regulator |  | 1.192 | 1.57E-02 | FXR1, PGK1, STIP1, UBB |
| CDK4/6 | group |  | 1.342 | 2.58E-04 | EIF5, FLNA, TPI1, TUBA1C, TUBB |
| CCND1 | transcription regulator |  | 1.342 | 9.38E-03 | EIF5, FLNA, SERF2, TPI1, TUBA1C, TUBB |
| OGA | enzyme |  | 1.414 | 6.29E-04 | ACTG1, ARPC4, CAV1, CNOT3, FLNA, HIPK2, TSPAN4, TUBA1A |
| 26s Proteasome | complex |  | 1.964 | 7.35E-03 | CTSD, H2AX, PRNP, PSMC4 |
| miR-122-5p | mature microRNA |  | 1.982 | 9.21E-04 | ALDOA, CS,EEF1A1, PKM |
| mir-122 | microRNA | Activated | 2 | 5.57E-03 | ALDOA, CS, PKM, SH3BGRL3 |
| let-7 | microRNA | Activated | 2.2 | 2.29E-03 | CDC34, HK2, HMGA1, PKM, SNRPC |
| IKZF1 | transcription regulator | Activated | 2.214 | 2.38E-03 | CAV1, GRN, JUP, MGLL, SGSH |
| EGLN | group | Activated | 2.236 | 1.20E-03 | FTL, HK2, PGAM1, PGAM4, TPI1 |

Supplementary Table 3. Key resources table

| **Antibody** | **Source, catalog number** | **Application** | **Dilution** |
| --- | --- | --- | --- |
| UBE2C | Abnova, H00011065-M01 | Western blot | 1:1000 |
| HIF-1α | GeneTex, GTX127309 | Western blot | 1:2000 |
| β-actin | Sigma, A5441 | Western blot | 1:5000 |
| **Reagent** | **Source, catalog number** | **Function** | **Concentration** |
| Cobalt (II) chloride (CoCl_2_) | Sigma, 232696 | HIF-1α inducer | 12.5~50 μM |
| **shRNA Target Sequence** | | | |
| Gene symbol | Sequence | Region | Score |
| UBE2C_clone1 | CCCTTACAATGCGCCCACAGT | CDS | TRC,# TRCN0000004241 |
| UBE2C _clone2 | GCCTGTCCTTGTGTCGTCTTT | 3UTR | TRC,# TRCN0000368994 |
| Abbreviations: CDS: Coding sequence; 3UTR: 3' untranslated regions | | | |

Supplement Table4.The primer sequences for amplifing *UBE2C*, *ALDOA*, *TPI1*, *PGK1*, *PGAM1*, *PGAM4*, *PKM* and *HIF-1α*

| Gene name | Sequence |
| --- | --- |
| UBE2C-F | CCGGTGGGCAAAAGGCTA |
| UBE2C-R | TCCATGGATGGTCCCTACCC |
| ALDOA-F | CAAATCCAAGGGCGGTGTTG |
| ALDOA-R | CGTCCTTCTTGTACTGGGCA |
| TPI1-F | CAGGAAGTTCTTCGTTGGGGG |
| TPI1-R | GCACAAACCACCTCGGTGTC |
| PKG1-F | GCTGGACAAGCTGGACGTTA |
| PKG1-R | TGGGACAGCAGCCTTAATCC |
| PGAM1-F | TGTCAAGCATCTGGAGGGTC |
| PGAM1-R | TGCATGGGCTTGATAGGCTT |
| PGAM4-F | CCAAGCATGTGGAGGGTCTC |
| PGAM4-R | CACACCGTCTCTTCATCCCC |
| PKM-F | CATTACCAGCGACCCCACAG |
| PKM-R | GACCTGCCAGACTTGGTGAG |
| HIF-1α-F | TATGAGCCAGAAGAACTTTTAGGC |
| HIF-1α-R | CACCTCTTTTGGCAAGCATCCTG |
